# Preliminary Study by Differential Scanning Calorimetric Analysis of Red Blood Cells in Peripheral Artery Disease Patients Treated with Cilostazol: Correlation with Improvements in Walking Distance

**DOI:** 10.3390/ph18010060

**Published:** 2025-01-07

**Authors:** Dénes Lőrinczy, Dorottya Szabó, László Benkő

**Affiliations:** 1 Department of Biophysics, Medical School, University of Pécs, Pécs Szigeti Str. 12, H-7624 Pécs, Hungary; 2 Department of Vascular Surgery, Medical School, University of Pécs, H-7624 Pécs, Hungary; dr.szabo.dorottya@pte.hu (D.S.); benko.laszlo@pte.hu (L.B.)

**Keywords:** cilostazol, peripheral arterial disease, intermittent claudication, DSC, human red blood cells

## Abstract

**Objective**: Peripheral artery disease (PAD) is a prevalent vascular condition characterized by arterial narrowing, which impairs blood flow and manifests as intermittent claudication, a pain or cramping sensation induced by physical activity or ambulation. Walking distance is a crucial clinical indicator of peripheral artery disease, and it correlates with the disease severity and risk of mortality. It reflects the severity of the disease, with reduced mobility indicating an increased risk of morbidity. It can also inform on the efficacy of the treatment. Cilostazol, a phosphodiesterase III inhibitor, has been demonstrated to enhance walking distance in patients with peripheral artery disease through the dilation of blood vessels and the inhibition of platelet aggregation. With this preliminary study, we aimed to elucidate other possible effects of cilostazol, specifically its influence on the structural properties of red blood cells. **Methods**: 10 patients (5 men, 5 women) with PAD were treated with cilostazol over a three-month period. Its biochemical effects on RBCs were determined using differential scanning calorimetry (DSC). Patient’s blood samples were collected at the start of treatment, then after two weeks, one month, two months, and three months of therapy. **Results**: The DSC analysis revealed shifts in thermal properties, including change in peak (melting or denaturation) temperature (**T_p_**) and calorimetric enthalpy (**ΔH_cal_**), which indicate significant structural changes in red blood cells. These thermal property changes correlated with clinical improvements in walking distance reported by patients. **Conclusions**: Our findings suggest that cilostazol induces substantial biochemical modifications in red blood cells, enhancing their functional properties and contributing to improved clinical outcomes. This study highlights the potential of differential scanning calorimetry as an adjunctive method for assessing the effectiveness of treatments for peripheral artery disease at the cellular level. However, further investigation with larger patient cohorts is required to confirm these initial results.

## 1. Introduction

Peripheral Artery Disease (PAD) is a circulatory disorder characterized by the narrowing of arteries, leading to reduced blood flow to the limbs [1,2]. It is a clinical manifestation of atherosclerosis, driven by risk factors such as tobacco use, diabetes, hypertension, hyperlipidaemia, age, family history, obesity, and a sedentary lifestyle. These factors initiate and accelerate atherosclerosis, resulting in arterial narrowing and diminished blood flow [3].

Patients with PAD often experience claudication symptoms, cramping pain during walking, referred to as intermittent claudication, which subsides with rest. In severe cases, PAD progresses to critical limb-threatening ischemia (CLTI), characterized by ischemic rest pain, ulcers, infections, and gangrene [1,2]. Beyond its localized effects, PAD is associated with a heightened risk of cardiovascular events, such as myocardial infarction and stroke, due to shared systemic risk factors [4]. The disease’s impact on physical mobility and cardiovascular health underscores the importance of effective management strategies to improve patient outcomes.

Management of PAD includes lifestyle modifications, pharmacological therapies, and surgical interventions, with a primary focus on reducing cardiovascular risk factors and alleviating symptoms [4]. Pharmacotherapy plays a central role in slowing the progression of arterial stenosis and minimizing ischemic complications. Agents such as statins (for cholesterol reduction), aspirin, clopidogrel, and cilostazol (for antiplatelet activity) are commonly employed [4].

Cilostazol, a phosphodiesterase III inhibitor, is recommended as a first-line therapeutic agent for symptomatic relief in PAD patients [5]. It enhances walking distance, improves quality of life, and addresses key aspects of PAD pathology by promoting vasodilation, inhibiting platelet aggregation, and improving endothelial function while reducing inflammatory markers [5,6,7]. Clinical studies and meta-analyses consistently confirm its efficacy in significantly increasing pain-free and maximum walking distances in patients with intermittent claudication [8,9]. Despite its demonstrated benefits, cilostazol use is not without risks. Common side effects include headache, diarrhea, dizziness, and palpitations, largely linked to its vasodilatory effects and associated increases in heart rate [10]. While these adverse effects are typically mild, they may affect patient adherence to treatment. In patients with pre-existing cardiac conditions, cilostazol’s vasodilatory and chronotropic effects may exacerbate arrhythmias or worsen heart failure symptoms [11]. For such individuals, careful monitoring or alternative therapies may be warranted. Cilostazol has also been associated with gastrointestinal discomfort and, in rare cases, bleeding complications, particularly when used alongside antiplatelet or anticoagulant medications [12].

The efficacy and safety of cilostazol depend on a careful balance of its benefits and risks. Patient-specific factors, such as age, comorbidities, and concomitant therapies, play a pivotal role in determining the suitability of cilostazol. Elderly patients and those with renal or hepatic impairments may require adjusted dosing to reduce the risk of adverse effects [13]. Patient education is equally important in ensuring adherence, as understanding the potential benefits and side effects can enhance compliance and improve clinical outcomes.

## 2. Aim of the Study

This study aimed to explore other mechanisms by which cilostazol may exert its effect. Specifically, we investigated the impact of cilostazol on the thermodynamic properties of red blood cells (RBCs) in PAD patients over a 3-month treatment period. Changes in the structural and functional properties of red blood cells have previously been shown to play a crucial role in PAD and the therapeutic effects of various drugs [14]. We utilized Differential Scanning Calorimetry (DSC), a technique that detects thermal transitions such as protein denaturation, to assess changes in the thermodynamic stability and structural properties of erythrocytes. We hypothesize that alterations in erythrocyte DSC profiles will correlate with observed clinical improvements, particularly, increased walking distance.

## 3. Patients and Methods

This preliminary study focused on patients with intermittent claudication, using the Fontaine classification to categorize their symptoms. The inclusion criteria required participants to be between the ages of 40 and 75, have a walking distance limitation of less than 400 m, and lack pedal pulses. Exclusion criteria included the presence of severe ischemic heart disease, chronic kidney failure, or limb-threatening ischemia.

In total, ten white adults (five men and five women, with a median age of 58.6 years) were enrolled in this preliminary study. The patients were observed for up to three months after enrolment, with one exception. A female participant required a femoral amputation on day 78 due to irreversible ischemic injury of her right foot. All participants were taking 100 mg of aspirin daily and statins at the start of the study, and their medications remained unchanged throughout the follow-up period.

Each patient underwent a supervised walking test administered by a physiotherapist. Before starting cilostazol therapy, control blood samples were collected from all patients. After a general physical examination, participants rested for 30 min before venipuncture, which was performed on the antecubital vein. Peripheral blood samples were collected from the study group (*n* = 5 in female and male group) and a healthy control group (*n* = 5 from both sexes). The treatment protocol involved the administration of 100 mg of cilostazol, taken twice daily. Patients attended clinical visits after 2 weeks 1 month, 2 months and 3 months. During these visits blood samples were collected, and their walking distances were evaluated. No adverse reactions to cilostazol were reported throughout the follow-up. The study received ethical approval from the Hungarian Medical Research Council (IV/2448-4/2022), and all participants provided written informed consent.

### 3.1. Blood Sample Preparation (RBCs)

Blood samples were collected into the special tubes (BD Vacutainer™ EDTA Tube) containing EDTA (1.5 mg/mL of blood). RBCs were separated by centrifugation at 1500× *g* for 15 min at 4 °C and washed three times with PBS buffer. The obtained RBCs were characterized by DSC. 

### 3.2. DSC Measurements

The thermal unfolding of human RBCs was analysed using a SETARAM Micro DSC-III calorimeter, following previously established protocols [15,16]. Experiments were performed within a temperature range of 0 to 100 °C, maintaining a consistent heating rate of 0.3 K/min. Denaturation studies utilized conventional Hastelloy batch vessels with an average sample volume of 950 μL, while 0.9% NaCl solution served as the reference sample. Sample and reference equilibration were conducted with a precision of ±0.1 mg. To correct the baseline, a repeated scan of the denatured sample was subtracted from the original DSC curve. Heat flow was plotted as a function of temperature, and thermal parameters were evaluated by calculating the calorimetric enthalpy from the area under the heat flow curve within the 40–90 °C range. This calculation employed the two-point peak integration feature of the SETARAM software. The thermal data are reported as averages ± standard deviations, with temperature values rounded to one decimal place and calorimetric enthalpy to two decimal places.

## 4. Results and Discussion

**Figure 1:**
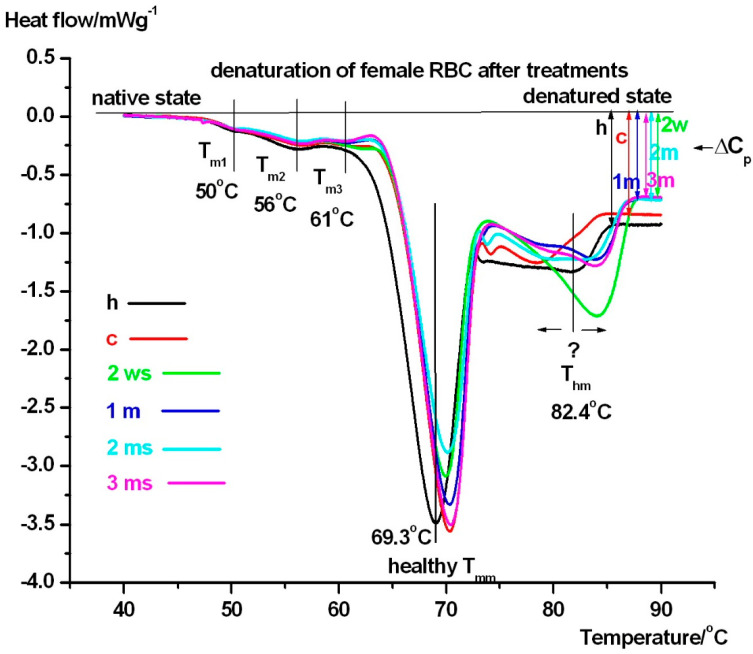
The presented heat flows of female RBCs (normalised on sample mass) are plotted in the function of sample temperature, involving a native state (no unfolding), the low thermal transitions (**T_m1_-T_m3_**), the main unfolding range (**T_mm_**) as well as a higher denaturation part (**T_hm_**) ending with a whole denatured state (symbols: **ΔCp** with arrows is the heat capacity change between the native and completely unfolded state. Healthy patient is signed by black solid line, and control (before treatment) with red. 2 weeks of treatment is represented by green line, 1 month is blue line, 2 months is cyan line, and 3 months is magenta solid line. (Endotherm deflections are downward).

In Figure 1. the average denaturation curves of RBCs can be seen from female patients including the healthy controls too (see comments in figure), while Table 1 demonstrates their main thermal parameters.

The denaturation of female RBCs exhibits three transitions: a lower denaturation range, where the formerly identified denaturation compounds [15,16,17,18,19,20,21] also appear prominently in our case too: spectrin, around 50 °C (**T_m1_**), bands of proteins 2.1, 4.1, and 4.2 around 56 °C (**T_m2_**), as well as the B3 glycoprotein at ~60–61 °C (**T_m3_**). In case of healthy female **T_m3_** is missing, and the calorimetric enthalpy (**ΔH_cal_**) of this range differs from each other in the range of the equipment precision of enthalpy detection (~5%, it is not listed in Table 1).

The middle part of RBC unfolding in healthy samples occurs at approximately 69 °C (Figure 1) and varies in the time duration of treatment between 69 and 71 °C. This might indicate a mild treatment time dependence. This stage corresponds to the melting of haemoglobin [18]. The half-width of unfolding (**T_mm1/2_**) decreases significantly after two weeks of treatment compared to healthy controls, potentially indicating a shock-like response to the starting of medical intervention. This may suggest that cilostazol binding makes haemoglobin’s structure more “compact, cooperative”. Furthermore, between one and three months of treatment, (**T_mm1/2_**) returns to healthy levels. This possibly indicates a steady state phenomenon in the treatment. Comparing to international literature data, the endotherms above 75 °C were surprising. We have observed this effect in case of guinea pigs [19,20], which we attributed to the binding of cyclophosphamide to RBC: the structure of the haemoglobin domain binding it became more rigid, this way more energy is needed for its unfolding, which manifested in a higher denaturation temperature. The course of the DSC curves in this temperature range showed a strong treatment time dependence. The self-control patient sample (red line) resulted in the lowest **T_hm_** (~78 °C)—compared with treated ones. The healthy female scan (**black line**) showed a higher **T_hm_** (~82 °C), while after two weeks of treatment (green line, like a shock effect of the treatment) we measured the highest **T_hm_** (~85 °C) with the highest enthalpy contribution (it is not shown in Table 1, but area under the green line represents it clearly).

**Figure 2:**
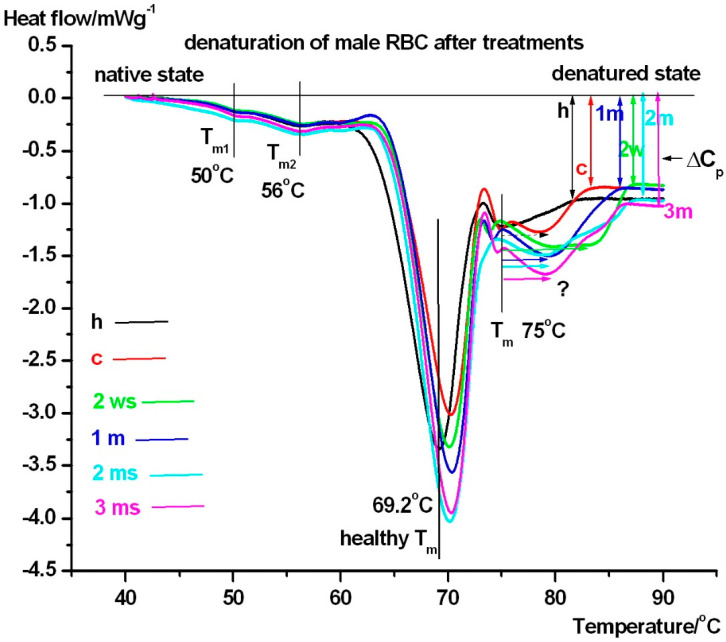
The presented DSC scans (normalised on sample mass) are plotted as a function of sample temperature. The meaning of the symbols/colours is the same as in case of Figure 1.

Another notable finding was the decrease in **ΔC_p_** at the different treatment times, compared to healthy controls. We believe this is a consequence of giving cilostazol to the patients. This reduction suggests a decreased aggregation capability of RBCs during treatment, potentially lowering the likelihood of thrombus formation. Figure 2 and Table 2 contain the thermodynamic data for male persons.

The denaturation of male red blood cells also exhibits three distinct transitions. The lower denaturation range is similar to that observed in females, with **T_m3_** also absent in case of healthy control. However, the **ΔH_cal_** in this range shows higher fluctuations compared to healthy controls (not shown in Table 2). The middle part of unfolding of RBC in case of all investigated samples and in the time duration of treatment varies between 69–71 °C, exhibiting a mild treatment time dependence. This refers to the melting of haemoglobin [18]. The half width of unfolding (**T_mm1/2_**) significantly decreased after 2 weeks treatment compared to the healthy control, which can be a shock-like answer on the medical treatment as in case of female’s RBCs, indicating that the binding of cilostazol to haemoglobin made its structure more “compact”. At the one- and three-months treatment timepoint, **T_mm1/2_** returns to healthy value, potentially indicating a steady-state effect in treatment. However, at two months, the highest **T_mm1/2_** value was observed, and this may suggest an internal loosening of haemoglobin structure, reflecting reduced cooperativity amongst its inner structural domains.

The endotherms in the higher denaturation range had significantly smaller **T_hm_** (except in case healthy and baseline-control) than in case of female patients. The course of the DSC curves in this temperature range showed a strong treatment time dependence too. The healthy-control patient sample (black line) resulted in the lowest **T_hm_** (~75 °C)—compared with treated ones. The scan at two weeks treatment showed a highest **T_hm_** (~81 °C), while after two weeks of treatment (like a shock effect of the treatment) with the highest enthalpy contribution (it is not shown in Table 2, but area under the red line represents it clearly).

**Figure 3:**
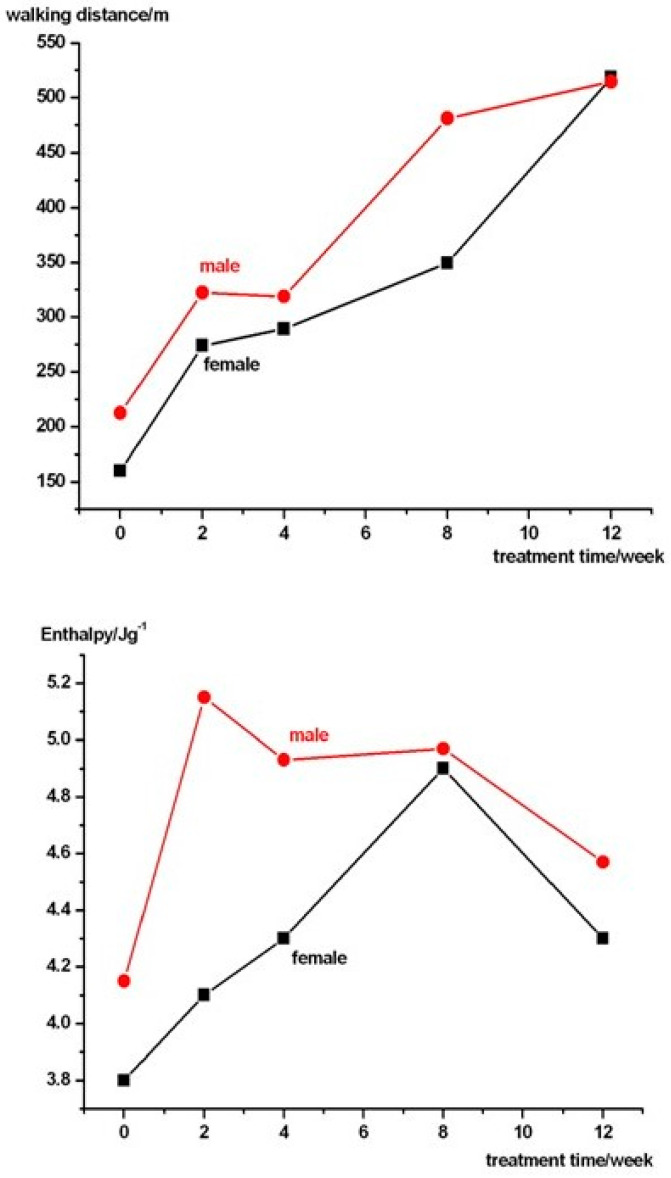
The average painless walking distance and enthalpy of the treated patients in both sexes.

In the male group, the decrease in **ΔC_p_** compared to healthy controls, is another consequence of cilostazol treatment. It differs from the pattern seen in females. It is only observed in male patients before and after two weeks and one month of cilostazol treatment. This suggests that sex may play a role in the physiological response to this drug. The Figure 3 and Table 3 represents the improvement of the painless walking distance in case of all treated patients.

As shown in Figure 3, the tendency for the pain-free waking distance to increase was more rapid at the beginning of the drug treatment for men, with the most significant difference observed after two months. However, this difference disappears by the third month.

Data in Table 3. reveal a considerable variation in the effects of the medical treatment, likely due to differences in the general health condition and drug sensitivity of the patients studied. This finding emphasises the need for medical doctors to consider alternative or more effective treatments at an earlier stage of PAD’s patient care.

## 5. Conclusions

The findings of this study, derived from DSC analysis, reveal that cilostazol treatment induces significant alterations in the thermal denaturation properties of red blood cells in patients with peripheral artery disease, suggesting critical biochemical modifications in protein stability and functionality. Three distinct denaturation transitions were identified, with key proteins such as spectrin, membrane proteins 2.1, 4.1, 4.2, and B3 glycoprotein displaying shifts in melting temperatures (**T_mm_** values). Notably, in female patients, the absence of the **T_m3_** transition in healthy RBCs was restored after cilostazol treatment, indicating enhanced stabilization of protein components near 60–61 °C. Temporal shifts in **T_mm_** values observed in both sexes further suggest that cilostazol modulates RBC protein structure and membrane dynamics over the treatment period.

The marked, shock-like reduction in the half-width of thermal unfolding (**T_mm1/2_**) after two weeks of cilostazol administration points to an acute structural compaction of hemoglobin, likely reflecting the drug’s impact on RBC protein organization. This effect appears to normalize over the three-month treatment period, indicative of an adaptive restructuring process within RBCs.

Sex-specific differences were prominent, with RBCs from male patients exhibiting lower thermal stability in the higher denaturation range compared to females. This distinction may point to divergent patterns of protein reorganization between sexes, potentially influencing RBC deformability, blood viscosity, and oxygen transport efficiency, all of which are critical for effective microcirculation in PAD.

Cilostazol-induced structural changes in RBCs, particularly hemoglobin compaction and reduced **ΔCp** values, likely contribute to enhanced RBC deformability and reduced aggregation. RBC compaction also influences platelet activity indirectly [22]. In PAD, elevated RBC aggregation and impaired deformability can promote platelet adhesion to the vessel walls, initiating thrombus formation [23]. By improving RBC stability and reducing aggregation, cilostazol may help minimize platelet activation. Furthermore, cilostazol’s known anti-thrombotic properties (mediated through cAMP elevation) likely complement these structural changes, further reducing platelet aggregation and lowering the risk of thrombus formation. These modifications collectively enhance peripheral circulation, mitigate microvascular obstruction, and reduce thrombotic risk, thereby augmenting cilostazol’s therapeutic benefits.

The observed spread in walking distance improvements among patients reflects a combination of RBC-related changes and systemic factors such as vascular and muscular health, which DSC measurements cannot fully capture. Improved RBC stability and deformability enhance microvascular perfusion, oxygen delivery, and tissue health, directly alleviating claudication symptoms. However, the variability in functional outcomes suggests that additional systemic factors play a role, which may explain the partial mismatch between DSC data and walking distance results.

These findings open new research avenues to better understand cilostazol’s biochemical effects on RBCs and its potential applications in personalized PAD treatments. Furthermore, the correlation between DSC thermal shifts and improved pain-free walking distances aligns with cilostazol’s known effects on enhancing blood flow and reducing inflammation, supporting DSC as a potential biomarker for evaluating cilostazol’s therapeutic impact in PAD patients. However, the significant variability observed in treatment responses suggests that further research is needed to fully understand the biochemical and clinical implications of cilostazol on RBC function.

## Figures and Tables

**Table 1:** Denaturation parameters of human female RBC in case of healthy control (green) and patients before (control: blue) and after the medical intervention (black), depending on the treatment time (the symbol * stands for significant difference compared healthy, while ^#^ is the same referring to control persons at *p* ˂ 0.05). The symbols: **T_mm_** is the middle denaturation temperature, **T_mm1/2_** stands for the temperature range at half value of its maximum heat flow, **T_hm_** and **T_hm1/2_** stands for the higher temperature range, and **ΔH_tcal_** represents the total calorimetric enthalpy of both last (middle and the higher temperature) range normalised on sample mass. Data are averages ± s.d. rounded to one decimal place for temperature and two decimal places for enthalpy.

Female (*n* = 5)	Thermodynamic Parameters				
	T_mm_/°C	T_mm1/2_/°C	T_hm_/°C	T_hm1/2_/°C	ΔH_tcal_/Jg^−1^
healthy	69.3 ± 0.4	4.2 ± 0.3	82.4 ± 0.4	8.5 ± 0.3	4.3 ± 0.20
control	70.3 ± 0.6 *	3.9 ± 0.2 *	78.0 ± 0.3 *	5.4 ± 0.2 *	3.8 ± 0.16 *
2 weeks	70.0 ± 0.8 *	3.6 ± 0.2 *	85.0 ± 0.2 *^#^	5.8 ± 0.2 *^#^	4.1 ± 0.18 ^#^
1 month	70.5 ± 0.9 *	4.1 ± 0.2 ^#^	85.0 ± 0.2 *^#^	7.0 ± 0.3 *^#^	4.3 ± 0.20 ^#^
2 months	70.5 ± 0.9 *	4.2 ± 0.3 ^#^	83.5 ± 0.3 *^#^	8.5 ± 0.4 ^#^	4.9 ± 0.21 *^#^
3 months	70.6 ± 0.7 *	4.2 ± 0.3 ^#^	84.5 ± 0.4 *^#^	7.3 ± 0.3 *^#^	4.3 ± 0.19 ^#^

**Table 2:** Denaturation parameters of human male RBCs in case of healthy control (black) and patients before (red) and after the medical intervention, depending on the treatment time. The symbols are the same as in case of Table 1. Data are averages ± s.d. (significance level at *p* ˂ 0.05 referring to healthy patients denoted by *, while for control by ^#^) rounded to one decimal place for temperature and two decimal places for enthalpy.

Male (*n* = 5)	Thermodynamic Parameters				
	T_mm_/°C	T_mm1/2_/°C	T_mh_/°C	T_mh1/2_/°C	ΔH_tcal_/Jg^−1^
healthy	69.2 ± 0.4	4.5 ± 0.1	75.9 ± 0.5	4.5 ± 0.2	3.92 ± 0.17
control	69.5 ± 0.6	4.1 ± 0.2 *	79.9 ± 0.6 *	7.0 ± 0.3 *	4.15 ± 0.19 *
2 weeks	69.4 ± 0.8	4.1 ± 0.3 *	81.3 ± 0.7 *^#^	8.2 ± 0.3 *^#^	5.15 ± 0.21 *^#^
1 month	69.6 ± 0.9	4.2 ± 0.3 *	80.0 ± 0.6 *	6.1 ± 0.2 *^#^	4.93 ± 0.20 *^#^
2 months	69.4 ± 0.9	4.7 ± 0.4 ^#^	79.8 ± 0.6 *	7.0 ± 0.3 *	4.97 ± 0.21 *^#^
3 months	69.6 ± 0.7	4.2 ± 0.6	79.9 ± 0.7 *	8.2 ± 0.4 *^#^	4.57 ± 0.18 *^#^

**Table 3:** The pain-free walking distance in case of treated patients. The red bold numbers indicate the sex dependence of the drug administration.

Samples (*n* = 5)	Painless Walking Distance/m			
	Female		Male	
	Average	s.d.	Average	s.d.
control	160	80	**212.5**	70.1
2 weeks	273.8	122.5	**322.3**	133.3
4 weeks	289.4	135.7	**319**	96.9
8 weeks	349.4	209.1	**481.3**	280.3
12 weeks	518.8	320.6	514.5	382.9

## Data Availability

There are no additional available data to upload.
